# CircPTP4A2 (hsa_circ_0007364) promotes growth and invasion of non-small cell lung cancer by regulating miR-183-5p/EEF2 axis

**DOI:** 10.1038/s41598-026-50751-4

**Published:** 2026-05-08

**Authors:** Qiuping Shen, Hao Jin, Xiaofeng Zhu

**Affiliations:** 1 Department of Medical oncology, Tongxiang First People’s Hospital, No. 1918 East School Road, Tongxiang, 314500 Zhejiang China; 2 Department of Cardiothoracic Surgery, Panjin Liaoyou Gem Flower Hospital, 26 Yingbin Road, Panjin, 124010 Liaoning China; 3 Department of Thoracic Surgery, Tongxiang First People’s Hospital, No. 1918 East School Road, Tongxiang, 314500 Zhejiang China

**Keywords:** CircPTP4A2, Non-small cell lung cancer, MiR-183-5p, EEF2, Invasion, Cancer, Genetics, Molecular biology

## Abstract

Recently, a novel circular RNA (circRNA), circPTP4A2 (hsa_circ_0007364), was reported to promote cervical cancer progression. However, its specific role in non-small cell lung cancer (NSCLC) remains unclear. Here, we observed that circPTP4A2 is significantly upregulated in NSCLC tissues and cell lines. Furthermore, multivariate Cox proportional hazards regression analysis confirmed that high circPTP4A2 expression is independently associated with poor prognosis in NSCLC patients after adjusting for confounding clinicopathological covariates. Knockdown of circPTP4A2 significantly suppressed the proliferation, migration, and invasion capabilities of H1299 and A549 cells. Additionally, we identified miR-183-5p as a direct targeted of circPTP4A2. In NSCLC clinical samples, circPTP4A2 expression showed a negative correlation with miR-183-5p levels. Moreover, we demonstrated that circPTP4A2 enhances the expression of EEF2, a downstream target of miR-183-5p. Collectively, our findings elucidate that circPTP4A2 promotes NSCLC progression by sponging miR-183-5p to upregulate EEF2, suggesting it could be a promising therapeutic target for NSCLC.

**Supplementary Information:**

The online version contains supplementary material available at 10.1038/s41598-026-50751-4.

## Introduction

 Lung cancer remains a prevalent malignancy that poses a significant threat to global health and is leading cause of mortality worldwide^1^. Projections for 2024 indicate that approximately 234,580 new cases (116,310 males and 118,270 females) of lung and bronchial cancer will be identified, resulting in 125,070 deaths (65,790 males and 59,280 females)^2^. The disease is primarily categorized into non-small cell lung cancer (NSCLC) and small cell lung cancer (SCLC), with NSCLC constituting about 85% of all cases^3^. The incidence of NSCLC continues to rise, a trend partially attributable to the worsening of air quality^4^. Although research on the pathogenesis, diagnosis, and treatment of NSCLC has advanced in recent years, the 5-year survival rate remains dismal due to rapid disease progression, particularly in patients with distant metastases^5^. The underlying pathogenesis of NSCLC is not fully understood, which impedes the development of more effective therapies. Fortunately, breakthroughs in identifying oncogenic mutations have paved the way for targeted therapies. Therefore, the discovery of novel targeted biomarkers will be crucial for improving the clinical treatment of NSCLC^6,7^.

Circular RNAs (circRNAs) are covalently closed-loop RNA molecules ubiquitously expressed in eukaryotic cells^8^. While the majority of circRNAs lack protein-coding ability, a specific subset retains the potential to be translated into peptides or proteins^9^. Accumulating evidence indicates that circRNAs are deeply implicated in the pathogenesis of human diseases, particularly malignancies. Functionally, they can serve as either oncogenes or tumor suppressors by sequestering microRNAs (miRNAs) or interacting with RNA-binding proteins (RBPs). Specifically, circPTP4A2 (hsa_circ_0007364) is generated via the backsplicing of 2 exons from the protein tyrosine phosphatase 4A2 (PTP4A2) gene^10^. Existing literature indicates that circPTP4A2 is aberrantly expressed in gliomas and potentially drives the progression of cervical cancer^11^. Beyond oncology, this circRNA is implicated in diverse pathological processes. For instance, it facilitates microglia polarization during cerebral ischemic stroke through the miR-20b-5p/YTHDF1/TIMP2 axis^12^. Despite these findings, comprehensive investigations into the function of circPTP4A2 in human cancer remain limited. While a recent study demonstrated that silencing circPTP4A2 can restrain NSCLC progression by modulating proliferation and stimulating anti-tumor immunity^13^, the detailed molecular mechanisms underlying these effects are still not fully elucidated. Given the versatile oncogenic properties of circPTP4A2 across various pathologies, we were prompted to investigate its functional significance in NSCLC. Nevertheless, its precise biological behaviors and the detailed molecular networks it orchestrates in this malignancy are yet to be fully defined. Consequently, this study aims to systematically characterize the contribution of circPTP4A2 to NSCLC progression and decode its downstream regulatory mechanisms.

MiR-183-5p is a well-documented miRNA frequently implicated in diverse malignancies. For instance, it drives breast cancer progression by targeting FHL1^14^ and modulates tumor cell apoptosis by controlling PNPT1 expression^15^. Furthermore, dysregulated protein elongation can trigger the activation of oncogenic signaling cascades, ultimately disrupting normal cellular homeostasis^16^. As a fundamental component of this process, eukaryotic elongation factor 2 (EEF2) serves as a vital regulator of protein synthesis by facilitating the translocation of ribosomes along mRNAs to extend peptide chains^17,18^. Consequently, abnormal EEF2 expression and impaired signal transduction can cause uncontrolled protein synthesis in malignant cells, accelerating tumor proliferation^16^. In the context of NSCLC, EEF2 has proven to be a critical functional mediator. Previous research established that EEF2 modulates NSCLC pathogenesis through specific pathways, such as the MALAT1-miR-515-5p-EEF2^19^ and PRMT7-HSPA5-EEF2 axes^20^. Nevertheless, whether EEF2 influences NSCLC progression specifically via a circRNA-miRNA-EEF2 regulatory network remains largely unexplored. Thus, clarifying the regulatory roles of miR-183-5p and EEF2 in NSCLC warrants further investigation.

In the current study, we sought to delineate the specific impact of circPTP4A2 on NSCLC pathogenesis. Our findings revealed a marked upregulation of circPTP4A2 in NSCLC clinical specimens compared to matched adjacent non-tumor tissues. Mechanistically, circPTP4A2 facilitates tumor cell proliferation and invasiveness by acting as a sponge for miR-183-5p, which consequently elevates EEF2 expression.

## Materials and methods

### Bioinformatic analysis

To screen for differentially expressed circRNAs (DEcRNAs) distinguishing NSCLC lesions from adjacent non-tumor tissues, three expression datasets (GSE158695, GSE101586, and GSE112214) were retrieved from the Gene Expression Omnibus (GEO) repository (https://www.ncbi.nlm.nih.gov/geo/). These profiles were generated utilizing the Agilent-069978 Arraystar Human CircRNA microarray V1 platform. Following the application of quantile normalization to the raw matrices, differential expression evaluation was executed via the ‘limma’ package within the R environment. To correct for multiple comparisons, P-values were modified via the Benjamini-Hochberg approach. Transcripts meeting the thresholds of an adjusted P-value < 0.05 and a |log2 fold change (FC)| > 1 were classified as DEcRNAs. Furthermore, bioinformatic forecasting of circPTP4A2-targeted miRNAs and miR-183-5p-targeted mRNAs was conducted utilizing the Circular RNA Interactome (https://circinteractome.nia.nih.gov) and starBase (http://starbase.sysu.edu.cn/index.php) platforms, respectively.

### Clinical samples

Paired tumor specimens and adjacent non-pathological tissues were surgically collected from 60 individuals pathologically confirmed with NSCLC (encompassing adenocarcinoma and squamous cell carcinoma) at Tongxiang First People’s Hospital. To be included in the study cohort, participants had to be adults (1 ≥ 8 years of age) undergoing radical resection, with a definitive NSCLC diagnosis based on the 8th edition of the lung cancer staging guidelines. Furthermore, all enrolled subjects were strictly treatment-naive prior to the operation and had no history of prior malignancies. Conversely, candidates were disqualified if they exhibited concurrent neoplasms, active infectious diseases, major organ failure, or pre-existing autoimmune conditions. Tissues were kept at −80 °C for subsequent analysis. Detailed clinical and pathological characteristics of patients with NSCLC are presented in Table 1. The patient’s survival time was recorded during a 60-month follow-up period. During the 60-month follow-up period, a total of 38 OS events occurred, and 3 patients were lost to follow-up. The median follow-up time was 42.5 months. This research was approved by the Ethics Committee of Tongxiang First People’s Hospital (approval number [TX-H-20230518], approved on May 18, 2023) and complied with the Declaration of Helsinki. Informed consent was obtained from all patients to publish any images, clinical data, and other information included in the manuscript, ensuring that the study complies with all regulations.

**Table 1:** Clinical pathological materials of 60 cases with NSCLC.

Variable	circPTP4A2 expression		*P* value
	Low (30)	High (30)	
Age			
< 60	13	14	0.795
≥ 60	17	16	
Sex			
Male	19	13	0.121
Female	11	17	
Tumor stage			
I + II	17	6	0.003^**^
III + IV	13	24	
Differentiation			
Well	15	18	0.436
Moderate-poor	15	12	
Lymph node metastasis			
Negative	13	5	0.024^**^
Positive	17	25	
Smoking status			
Yes	14	17	0.438
No	16	13	

### Cell culture and treatment

Human bronchial epithelial cells (Beas-2B, Cat. CRL-3588) and three NSCLC cell lines H1299 (Cat. CRL-5803), A549 (Cat. CCL-185), and H460 (Cat. HTB-177), were procured from the American Type Culture Collection (ATCC, USA). To verify their genetic integrity, short tandem repeat (STR) analysis was conducted on all cultures within the past three years. Additionally, routine screening for mycoplasma was carried out on a monthly basis, and all cells were validated as mycoplasma-free prior to any assays using a PCR-based Mycoplasma Detection Kit (Cat. ab289834, Abcam, USA). All cell cultures were propagated in Roswell Park Memorial Institute-1640 (RPMI-1640; Hyclone, USA) enriched with 10% fetal bovine serum (FBS, Gibco, USA) and 1% penicillin-streptomycin. The incubation environment was constantly maintained at 37 °C with 5% CO2 and standard humidity. To prevent phenotypic alterations, only cells spanning passages 5 through 15 were subjected to downstream functional evaluations. To block transcription, Actinomycin D (2 mg/mL; Sigma-Aldrich, USA) was administered to A549 and H1299 cells. For RNA stability verification, the extracted total RNA was digested using 3 U/mg RNase R (Epicentre, USA) for 15 min at 37 °C.

### Quantitative real-time polymerase chain reaction (qRT-PCR)

Total RNA isolation was performed with Trizol reagent (Invitrogen), followed by reverse transcription into complementary DNA (cDNA) utilizing the PrimeScript RT Reagent Kit (Takara). Subsequent qRT-PCR assays were executed employing a SYBR Green mixture (Vazyme). Relative transcript abundance was determined via the standard 2^−ΔΔCt^ approach^21^, where GAPDH served as the endogenous reference for circPTP4A2 and EEF2, while U6 was utilized for miR-183-5p normalization.

The specific primer sequences:

CircPTP4A2:

Forward: 5’-GGAGTGACGACTTTGGTTCG-3’,

Reverse: 5’-TGTCAGCGAAAATGCTGTGC-3’;

miR-183-5p:

Forward: 5’-CCTGTTCTGTGTATGGCACTGGT-3’,

Reverse: 5’-TTCACTGACTGAGACTGTTCACAGTG-3’;

EEF2:

Forward: 5’-GCCTCATGGAGCCCATCTAC-3’,

Reverse: 5’-AGGGATGCCTTCTTTCAGGC-3’;

U6:

Forward: 5’-CTCGCTTCGGCAGCACA-3’,

Reverse: 5’-AACGCTTCACGAATTTGCGT-3’;

GAPDH:

Forward: 5’-CCAGGTGGTCTCCTCTGA-3’,

Reverse: 5’-GCTGTAGCCAAATCGTTGT-3’.

### Cell transfection

All small interfering RNAs (siRNAs) targeting circPTP4A2, miR-183-5p mimics and inhibitors, along with their corresponding negative controls (si-NC, miR-NC mimics, and miR-NC inhibitors), were commercially synthesized by GenePharma (Shanghai, China). For stable knockdown, lentiviral vectors (pGLV3-H1-GFP-Puro; LV-circ1) against circPTP4A2 and control vectors (LV-NC) were constructed by GenePharma (Suzhou, China). Transfection into H1299 and A549 cells was facilitated using Lipofectamine 3000 (Invitrogen, CA, USA) following the provided commercial protocol. Detailed sequences for all oligonucleotides applied in these assays are summarized in Table S1.

### Fluorescence in situ hybridization (FISH) and Immunofluorescence (IF)

To assess the spatial distribution of circPTP4A2 and EEF2, a combined FISH and IF methodology was applied. The Fluor 488-conjugated probe specific to circPTP4A2 was customized by GenePharma (Shanghai, China), while a target-specific primary antibody was utilized for EEF2 detection. In short, H1299 and A549 cells seeded on glass coverslips underwent fixation in 4% paraformaldehyde, permeabilization with 0.5% Triton X-100, and subsequent blocking using 5% BSA. Hybridization with the circPTP4A2 probe was conducted overnight at 55 °C. Following thorough washes, specimens were probed with the anti-EEF2 antibody (1:200) at 4 °C overnight, and then exposed to a Cy3-labeled secondary antibody (1:500) for 1 h at ambient temperature. Nuclear counterstaining was achieved using DAPI (10 min). Confocal images were acquired via a Leica SP8 laser scanning microscope. For co-localization assessment, Pearson’s correlation coefficient (PCC) was computed utilizing the JACoP plugin within ImageJ to quantify the signal overlap between circPTP4A2 (green) and EEF2 (red).

### Cell proliferation assays

Cell Counting Kit-8 (CCK-8) and colony formation assays were utilized to assess the proliferative capacity of H1299 and A549 cells. For the CCK-8 test, transfected cells (2000 cells/well) were seeded into 96-well plates and cultured for 24, 48, and 72 h. Afterward, 10 µL of CCK-8 solution (Dojindo) was added to each well. After 2 h, the absorbance at 450 nm was measured via a microplate reader. For the colony formation assay, approximately 500 transfected A549 and H1299 cells were plated in 6-well plates. Seven days later, following staining with crystal violet solution, the colonies were counted using an Olympus camera.

### Transwell assay

Transwell assays were conducted in 24-well plates, with or without Matrigel (Corning) coating in the Transwell chamber. H1299 and A549 cells were seeded into the upper chamber (Corning, 8.0 μm pore size) in serum-free medium. The lower chamber, containing culture medium supplemented with 20% FBS, served as a chemoattractant. Cells were then incubated for 24 h for the migration assay or 48 h for the invasion assay. Next, the cells that migrated to the underside of the insert were fixed in 100% methanol, stained with 0.1% crystal violet, and photographed under an Olympus microscope. Five random fields were selected to count the number of cells, and the average values were calculated.

### Animal experiments

To investigate the impact of circPTP4A2 on tumor growth in vivo, A549 cells were stably transfected with LV-circ1 or LV-NC. Subsequently, these cells (5 × 10^6^) were subcutaneously injected into the flanks of 12 male BALB/c nude mice (4 weeks old; weighing 15–20 g). The animals were randomly assigned to two groups (*n* = 6 per group). They were maintained under controlled conditions (23 ± 2 °C, 55 ± 15% humidity, 12-hour light/dark cycle) with free access to food and water. Tumor dimensions were measured weekly using a digital caliper, and tumor volumes were calculated using the formula: Volume = π/6 × length × width^2^. Day 7 served as the baseline evaluation time point, at which no significant difference in tumor burden was noted between the groups. On day 28, the animals were anesthetized using vaporized isoflurane (1.5% concentration) and subsequently euthanized via cervical dislocation. Death was confirmed by the cessation of heartbeat and respiration, after which the tumor tissues were collected and weighed. Statistically, differences in final tumor weights were evaluated using an unpaired two-tailed Student’s t-test, while tumor growth curves (tumor volumes over time) were analyzed using a two-way ANOVA. This animal experiment was carried out in compliance with the ARRIVE guidelines and was approved by the Animal Ethics Committee of Tongxiang First People’s Hospital. All procedures were executed following the relevant regulations.

### Biotinylated RNA pull-down assay

The RNA pull-down assay was conducted following previously established protocols^22,23^. Briefly, to isolate miRNAs interacting with circPTP4A2, probe-coated magnetic beads were generated by incubating biotinylated circPTP4A2 probes with C-1 magnetic beads (RiboBio). Subsequently, the probe-bound beads and sonicated H1299 and A549 cell lysates were co-incubated overnight at 4 °C, followed by elution and qRT-PCR analysis.

### Dual‑luciferase reporter assay

The circPTP4A2 sequences containing wild-type or mutant miR-183-5p binding sites were synthesized and subsequently cloned into the psi-CHECK2 vector (Promega). Thereafter, the wild-type or mutant vectors, along with miR-183-5p mimics or mimic controls, were co-transfected into H1299 and A549 cells using the Lipofectamine 3000 reagent (Invitrogen). 48 h post-transfection, luciferase activities were evaluated via the dual-luciferase reporter assay system (Promega).

### RNA immunoprecipitation (RIP) assay

The EZ Magna RIP kit (17–701, Merck Millipore) was utilized for the RIP assay. Briefly, H1299 and A549 cells were scraped and collected in RIP lysis buffer supplemented with protease and RNase inhibitors. The resulting cell lysates were incubated overnight at 4 °C with antibodies directed against Ago2 or control IgG (CST). Subsequently, the magnetic beads were washed and digested with proteinase K to degrade bound proteins. Finally, the co-precipitated RNA was extracted and quantified via qRT-PCR.

### Western blot analysis

Total proteins were isolated using Radioimmunoprecipitation assay (RIPA) lysis buffer (Beyotime) and quantified with a BCA assay kit (Beyotime). The protein extracts were separated via Sodium dodecyl sulfate-polyacrylamide gel electrophoresis (SDS-PAGE) and subsequently electro-transferred onto polyvinylidene difluoride (PVDF) membranes (Merck KGaA). Thereafter, the membranes were incubated with specific primary antibodies at 4 °C overnight. The primary antibodies employed included those against EEF2 (1:500, Abcam, USA), PCNA (1:200, Cell Signaling Technology, USA), Cyclin D1 (1:2000, Abcam, USA), Bcl-2 (1:2000, Abcam, USA), Caspase-3 (1:1000, Cell Signaling Technology, USA), Bax (1:2000, Abcam, USA), and GAPDH (1:2000, Proteintech, USA). Following TBST washes, the blots were incubated with a secondary antibody (goat anti-mouse IgG, 1:2000, Proteintech, USA) for 2 h at room temperature. Finally, after three additional washes, the protein bands were visualized using an ECL reagent (Beyotime). The resulting signals were analyzed via ImageJ software and normalized to the GAPDH internal control.

### Statistical analysis

Statistical analyses were performed using SPSS 21.0 (IBM, NY, USA). Student’s t-test (unpaired) was used to evaluate statistical significance between two groups, while paired samples use paired t-tests. One-way or two-way ANOVA was used for comparisons among multiple groups. Pearson correlation analysis was performed to evaluate the correlation between two variables. The cut-off value for distinguishing high and low circPTP4A2 expression was determined using the median expression level of the cohort. Univariate and multivariate Cox proportional hazards regression models were used to evaluate the association between circPTP4A2 expression and patient survival outcomes. Covariates with a P-value < 0.05 in the univariate analysis were subsequently included in the multivariate Cox regression model to identify independent prognostic factors. Prior to conducting the multivariate Cox regression analysis, the proportional hazards assumption was statistically verified for all included covariates using Schoenfeld residuals. Survival curves were estimated using the Kaplan-Meier method and compared using the log-rank test. *P* < 0.05 was considered statistically significant.

## Results

### CircPTP4A2 expression is upregulated in NSCLC tissues

**Fig. 1:**
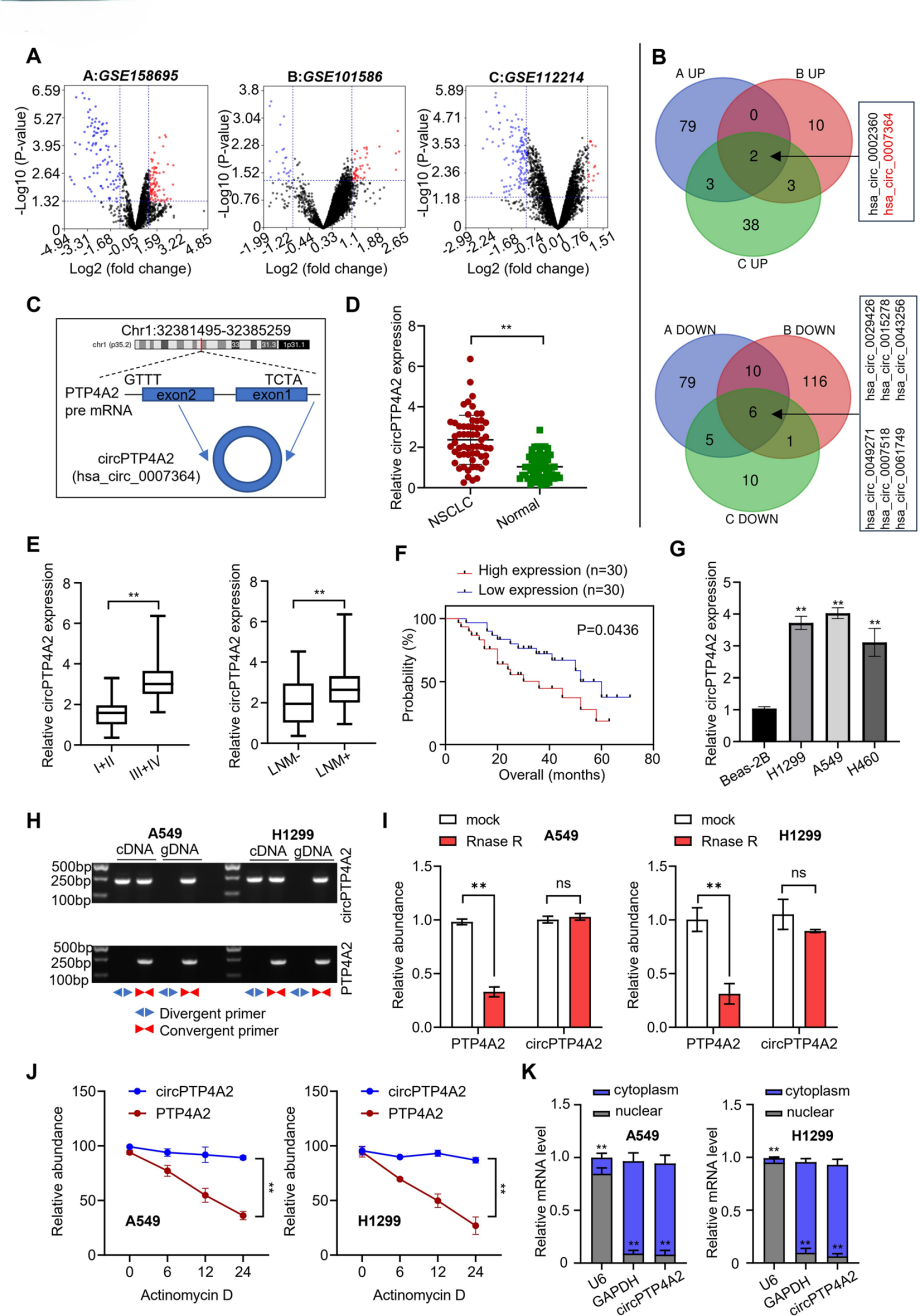
CircPTP4A2 expression is upregulated in NSCLC tissues. **A** Volcano plots of differentially expressed circRNAs from three GEO databases (GSE158695, GSE101586, and GSE112214). |Log2 FC| >1, adjusted *P* < 0.05 were used as the cutoff criteria. Log2 FC > 1 and adjusted *P* < 0.05 indicate upregulated circRNAs (red dots); Log2 FC < − 1 and adjusted *P* < 0.05 indicate downregulated circRNAs (blue dots). **B** Venn Diagram of differentially expressed circRNAs in NSCLC tissues based on the GEO databases. **C** Schematic model of circPTP4A2 formation. **D** CircPTP4A2 expression in NSCLC tumor tissues (*n* = 60) and adjacent normal tissues (*n* = 60) was examined by qRT-PCR. **E** CircPTP4A2 expression in patients with NSCLC with tumor stage and lymph node metastasis. **F** Kaplan-Meier analysis of the association of circPTP4A2 expression with overall survival in patients with NSCLC. **G** The expression of circPTP4A2 was detected in NSCLC cells (H1299, A549, H460) and human bronchial epithelial cell (Beas-2B) by qRT-PCR. **H** The representative agarose gel image showing the linear or back-splicing products amplified using convergent and divergent primers of NSCLC cell lines. **I** qRT-PCR results showing RNase R digestion of *PTP4A2* mRNA in H1299 and A549 cells. **J** The resistance of circPTP4A2 and *PTP4A2* mRNA to Actinomycin D was analyzed through qRT-PCR in A549 and H1299 cells. **K** Subcellular localization of circPTP4A2 in H1299 and A549 cell lines confirmed by cytosolic/nuclear fractionation assay. *N* = 3. **P < 0.01.

We first analyzed circRNA expression profiles from three GEO datasets (GSE158695, GSE101586, and GSE112214) to identify potential oncogenic circRNAs. The differential expression analysis for each dataset is visualized in the volcano plots (Fig. 1A) and heatmaps (Figure S1A) using the “ggplot2” (version 3.3.5; https://ggplot2.tidyverse.org/) and the “pheatmap” R package (version 1.0.12; https://cran.r-project.org/web/packages/pheatmap). Subsequently, Venn diagram analysis was performed to identify the overlapping differentially expressed circRNAs (DEcRNAs) across these three datasets. Among the upregulated candidates shown in the heatmap (Fig. 1B), hsa_circ_0007364 (circPTP4A2) was selected for further study due to its significant upregulation. According to the UCSC Genome Browser, circPTP4A2 is located at the 1p35.2 chromosomal region and spans 782 bases. It is formed through the back-splicing of exon1 and exon 2 of the *PTP4A2* pre-mRNA (Fig. 1C). Subsequently, we quantified circPTP4A2 expression in 60 pairs of NSCLC tissues and matched adjacent non-tumor tissues using qRT-PCR. As illustrated in Fig. 1D, the expression of circPTP4A2 was significantly upregulated in NSCLC samples compared to adjacent tissues (Fig. 1D, *P* < 0.01). Furthermore, the 60 patients were stratified based on tumor stage. Compared to those with stage I-II disease, circPTP4A2 levels were markedly elevated in stage III-IV NSCLC patients (Fig. 1E, left, *P* < 0.01). Likewise, circPTP4A2 expression was notably upregulated in NSCLC patients presenting with lymph node metastasis relative to those without such metastasis (Fig. 1E, right, *P* < 0.01). We subsequently explored the association between circPTP4A2 levels and patient prognosis. Using the median circPTP4A2 expression from the NSCLC cohort (Fig. 1D) as a threshold, the 60 cases were divided into high-expression (*n* = 30) and low-expression (*n* = 30) groups. Kaplan-Meier survival curves revealed that individuals in the high-expression group experienced a markedly poorer survival rate (*P* = 0.044, Fig. 1F). Furthermore, the associations between circPTP4A2 expression and standard clinicopathological features were evaluated (Table 1). A univariate Cox proportional hazards regression was then conducted to determine potential prognostic indicators in the NSCLC cohort. The findings revealed that tumor size, histological differentiation, lymph node metastasis, TNM stage, and circPTP4A2 expression emerged as significant prognostic variables (Table 2). These significant factors were subsequently incorporated into a multivariate analysis for further adjustment. Moreover, the multivariate Cox proportional regression model verified that elevated circPTP4A2 expression acts as an independent risk factor for poor overall survival in the NSCLC cohort (Table 2). Our findings demonstrated that circPTP4A2 was abundantly expressed in NSCLC cases presenting with advanced clinical stage (III + IV) and lymph node metastasis. Nevertheless, no meaningful associations were observed between circPTP4A2 expression and remaining clinical variables, including patient age, sex, smoking history, or histological differentiation (Table 1). In summary, our findings demonstrate that circPTP4A2 is markedly overexpressed in NSCLC tissues, correlating with an unfavorable prognosis in affected patients. To explore its expression patterns further, we evaluated circPTP4A2 abundance in NSCLC cell lines and normal human bronchial epithelial cells. Notably, circPTP4A2 was prominently upregulated in the test NSCLC cells, including H1299, A549, and H460 (Fig. 1G, *P* < 0.01). Guided by these expression profiles, H1299 and A549 cells were chosen for subsequent experiments.

**Table 2:** Univariate and multivariate Cox regression analyses of overall survival in NSCLC patients. Footnote: HR, hazard ratio; 95% CI, 95% confidence interval. Variables that were statistically significant in the univariate analysis (*P* < 0.05), including tumor size, histological differentiation, lymph node metastasis, TNM stage, and circPTP4A2 expression, were included in the multivariate Cox regression model.

Parameter	Univariate analysis			Multivariate analysis		
	HR	95%CI	*P*	HR	95%CI	*P*
Age (< 60 vs. ≥ 60)	1.463	0.624 ~ 3.429	0.381			
Gender (Male vs. Female)	0.976	0.417 ~ 2.286	0.955			
Tumor size (< 4 cm vs. ≥ 4 cm)	0.214	0.080 ~ 0.574	0.002*	0.089	0.007 ~ 1.210	0.069
Histological differentiation (well+moderately Vs poorly)	0.266	0.110 ~ 0.640	0.003*	0.102	0.030 ~ 0.347	<0.001*
Lymph node metastasis (negative vs. positive)	0.351	0.128 ~ 0.799	0.014*	0.428	0.074 ~ 1.021	0.078
TNM stage (I + II vs. III + IV)	0.327	0.134 ~ 0.799	0.014*	0.886	0.184 ~ 4.268	0.880
circPTP4A2 (low vs. high)	0.345	0.139 ~ 0.856	0.022*	0.159	0.032 ~ 0.782	0.024*

To exclude the potential for trans-splicing or genomic rearrangements, we conducted two assays to confirm the authentic head-to-tail splicing of circPTP4A2. Initially, convergent and divergent primers were synthesized to amplify the linear *PTP4A2* transcript and circPTP4A2 using cDNA and genomic DNA (gDNA) derived from A549 and H1299 cells. As illustrated in Fig. 1H, circPTP4A2 was exclusively amplified by divergent primers from the cDNA template, but not from gDNA (Fig. 1H). Furthermore, RNase R treatment demonstrated that circPTP4A2 exhibited strong resistance to exonuclease degradation relative to the linear *PTP4A2* mRNA (Fig. 1I, *P* < 0.01). Likewise, Actinomycin D exposure indicated that the half-life of circPTP4A2 was markedly extended compared with the linear isoform (Fig. 1J, *P* < 0.01). Subsequently, we assessed the subcellular distribution of circPTP4A2 in H1299 and A549 cells via a nuclear/cytosolic fractionation assay. The results indicated that circPTP4A2 is predominantly enriched in the cytoplasm of both cell lines (Fig. 1K, *P* < 0.01).

### CircPTP4A2 promotes NSCLC cell proliferation and metastasis

**Fig. 2:**
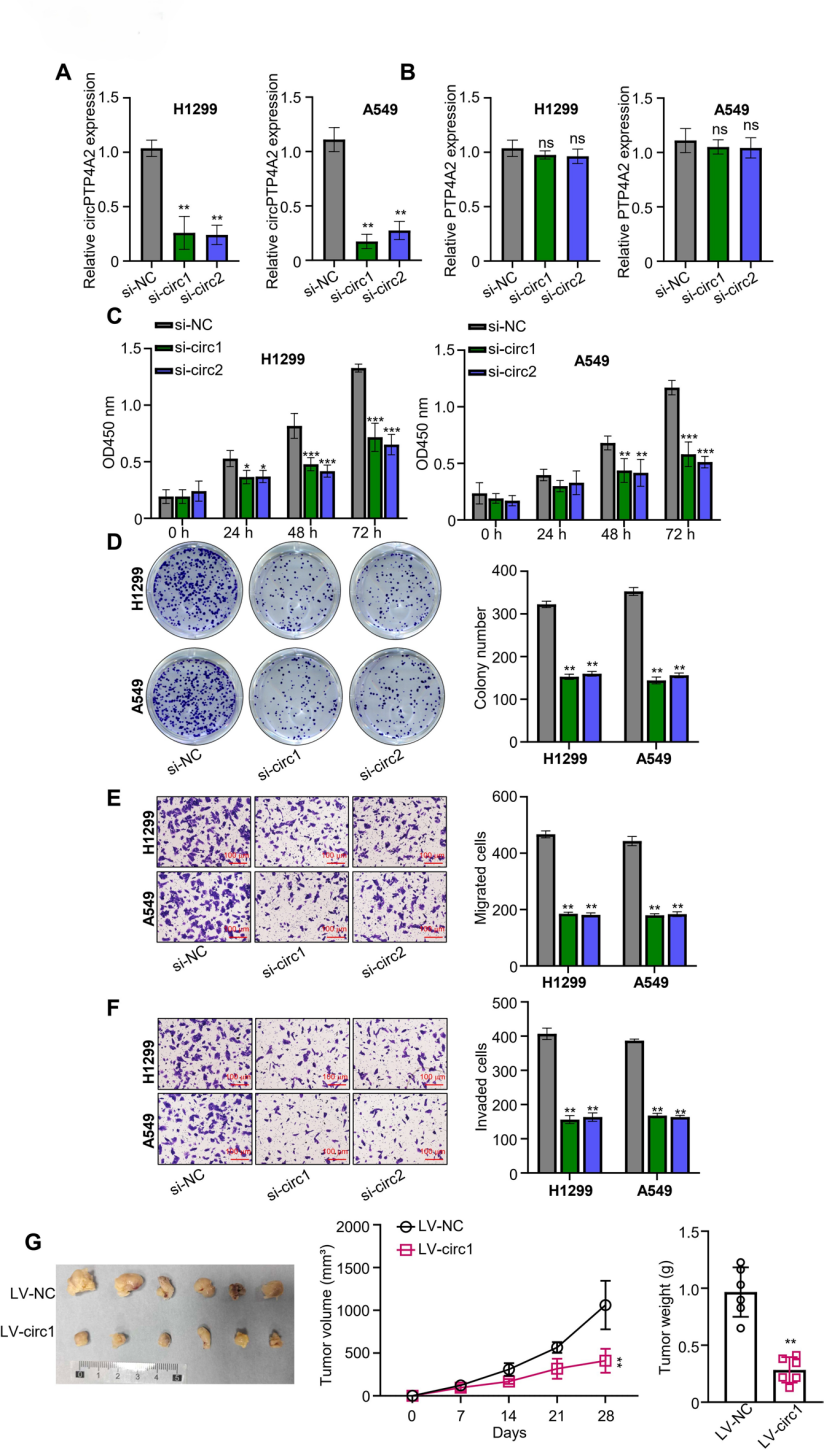
CircPTP4A2 is highly expressed in NSCLC cell lines and promotes proliferation and metastasis. **A** qRT-PCR analysis of circPTP4A2 expression after the transfection with si-circPTP4A2 in H1299 and A549 cells lines. **B** qRT-PCR analysis of *PTP4A2* expression after the transfection with si-circPTP4A2 in H1299 and A549 cells lines. **C** CCK-8 analysis of cell proliferation after transfection with si-circPTP4A2 in H1299 and A549 cells lines. **D** Colony formation analysis of cell colony number after transfection with si-circPTP4A2 in H1299 and A549 cells lines. **E** Transwell assay was conducted to evaluate the migration of H1299 and A549 cells (×200 magnification). **F** Transwell assay was conducted to evaluate the invasion of H1299 and A549 cells (×200 magnification). **G** Evaluation of tumor growth curves and tumor weights in nude mice following the subcutaneous injection of A549 cells stably transfected with LV-circ1 or LV-NC. Mice were randomly assigned to two groups (*n* = 6 per group), with day 7 established as the baseline for tumor measurement. Data are presented as mean ± SD. Statistical significance was determined using two-way ANOVA for tumor growth curves and an unpaired Student’s t-test for final tumor weights. **P < 0.01.

The observed upregulation of circPTP4A2 in NSCLC tissues and cell lines implies that it may exert an oncogenic role in this disease. To test this hypothesis, we designed siRNA specifically targeting circPTP4A2. As depicted in Fig. 2A, two independent siRNAs (si-circ1 and si-circ2) effectively knocked down the expression of circPTP4A2 in H1299 and A549 cells (Fig. 2A, *P* < 0.01). Furthermore, to validate the specificity of our knockdown strategy, we confirmed that the siRNAs targeting the back-splice junction had no noticeable impact on the linear *PTP4A2* mRNA expression (Fig. 2B). Subsequent functional assays demonstrated that circPTP4A2 silencing markedly suppressed cell proliferation, as evidenced by CCK-8 (Fig. 2C, *P* < 0.05) and colony formation assays (Fig. 2D, *P* < 0.01). Moreover, Transwell assays revealed that silencing circPTP4A2 markedly impaired the migratory and invasive capacities of H1299 and A549 cells (Fig. 2E and F, *P* < 0.01). To assess the in vivo role of circPTP4A2 in NSCLC progression, we established a xenograft mouse model by subcutaneously injecting A549 cells stably transfected with LV-circ1 into nude mice. Consequently, both tumor volumes and weights in the LV-circ1 group were significantly lower than those in the LV-NC group (Fig. 2G, *P* < 0.01). To verify the in vivo silencing efficacy, circPTP4A2 expression was quantified in the resected xenografts. The results confirmed that circPTP4A2 levels were persistently downregulated in the LV-circ1 group (Figure S1B, *P* < 0.01), confirming the stability of the knockdown. To further explore the molecular alterations underlying this sustained depletion, we examined the expression of key proliferation- and apoptosis-related proteins in the harvested tumor tissues via Western blotting (Figure S1C, *P* < 0.01). In alignment with the observed tumor growth inhibition, the protein levels of PCNA and Cyclin D1 were significantly reduced in the LV-circ1 group compared to the LV-NC control. Moreover, Bax expression was markedly increased, accompanied by a decrease in Bcl-2, leading to a significantly elevated Bax/Bcl-2 ratio. This pro-apoptotic transition was further corroborated by the upregulation of cleaved caspase-3. Taken together, these molecular data demonstrate that continuous knockdown of circPTP4A2 in vivo suppresses proliferation and promotes apoptosis, thereby providing mechanistic insight into the reduced tumor growth observed in the xenograft model. Ultimately, these findings substantiate that circPTP4A2 can acts to drive the progression of NSCLC.

### CircPTP4A2 acts as a sponge of miR-183-5p in NSCLC cells

**Fig. 3:**
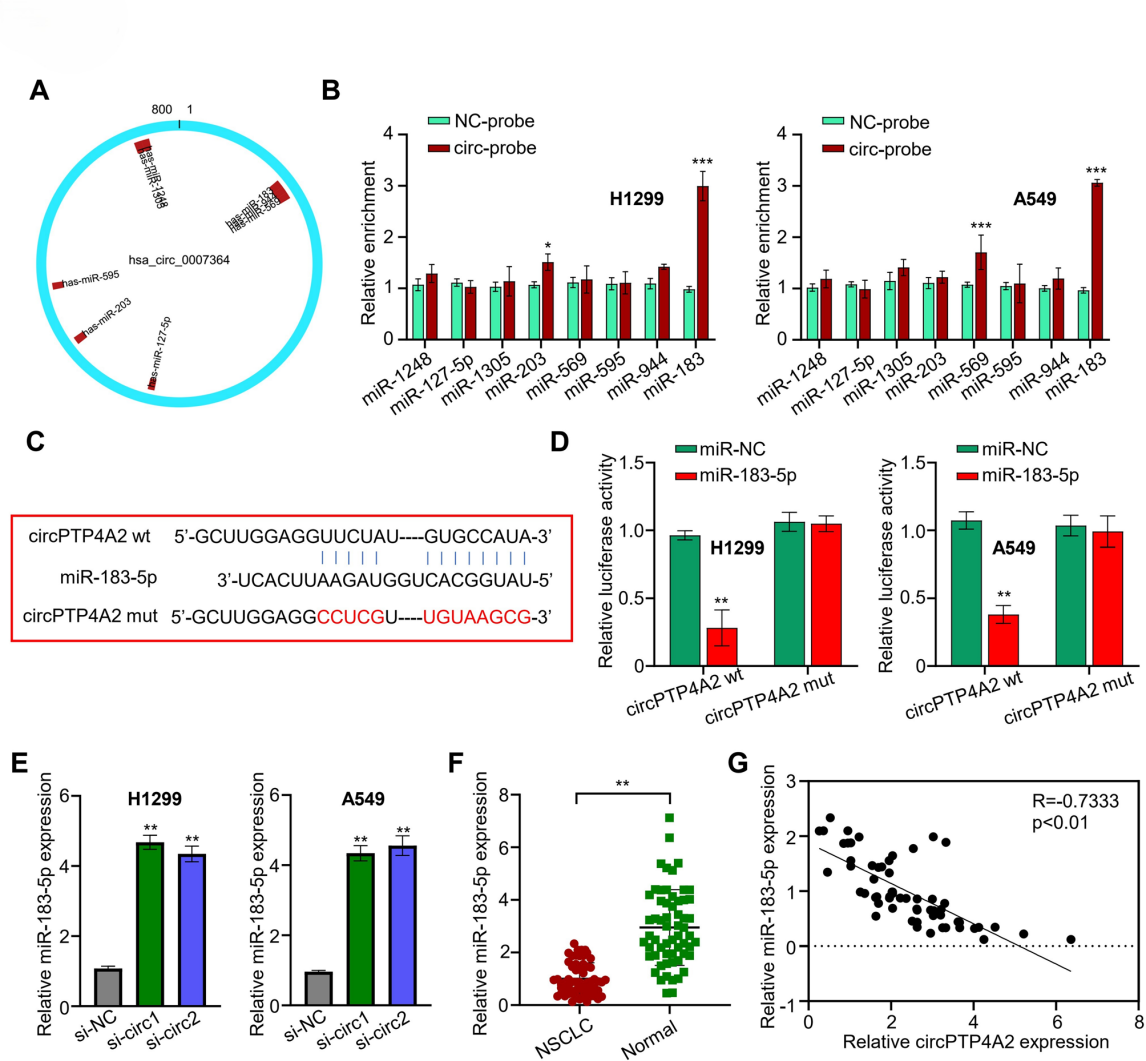
CircPTP4A2 is directly targeted by miR-183-5p. **a** Bioinformatics analysis to predict the putative binding sites of miR-183-5p in circPTP4A2. **b** RNA-pull down assay was carried out to confirm the target relationship between miRNAs and circPTP4A2. **c** Schematic representation of the binding sites of miR-183-5p with the 3′UTR of WT or Mut circPTP4A2. **d** Dual-luciferase reporter assay was used to assess the effect of miR-183-5p on circPTP4A2 in H1299 and A549 cells lines. **e** qRT-PCR analysis of the expression levels of miR-183-5p after transfection with si- circPTP4A2 in H1299 and A549 cells lines. **f** qRT-PCR analysis of miR-183-5p expression in lung cancer tissues (*n* = 60). **g** The Pearson’s correlation coefficients were used to evaluate the correlation between circPTP4A2 and miR-183-5p in lung cancer tissues (*n* = 60) (*r* = − 0.7333, *p* < 0.01). *N* = 3. **P < 0.01, ***P < 0.01.

To explore the underlying mechanism of circPTP4A2 in NSCLC cells, we predicted the potential binding of eight candidate miRNAs to the circPTP4A2 sequence using the CircInteractome database (Fig. 3A) prioritizing those with high context + score percentiles. Subsequently, we conducted an RNA pull-down assay using biotinylated circPTP4A2 probes to verify these putative interactions. The results revealed that among the candidates, only miR-183-5p was significantly enriched by circPTP4A2 (Fig. 3B, *P* < 0.05). Figure 3C illustrates the predicted binding site of miR-183-5p within the wild-type or mutate circPTP4A2 sequences (Fig. 3C). To further verify the interaction between circPTP4A2 and miR-183-5p, we performed dual-luciferase reporter assays. The results demonstrated that miR-183-5p mimics significantly attenuated the luciferase activity of the wild-type circPTP4A2 reporter in H1299 and A549 cells, but failed to suppress the activity of the mutant circPTP4A2 reporter (Fig. 3D, *P* < 0.01). Furthermore, qRT-PCR analysis revealed that silencingcircPTP4A2 elevated the levels of miR-183-5p in both H1299 and A549 cells (Fig. 3E, *P* < 0.01). In accordance with these findings, qRT-PCR evaluation revealed that miR-183-5p abundance in NSCLC specimens was markedly diminished compared to adjacent normal tissues (Fig. 3F, *P* < 0.01). Furthermore, an inverse correlation was observed between the expression of circPTP4A2 and miR-183-5p levels within the NSCLC cohort (*r* = − 0.7333, *P* < 0.01) (Fig. 3G). Taken together, these data substantiate the direct interaction between circPTP4A2 and miR-183-5p in the context of NSCLC.

### MiR-183-5p directly interacts with EEF2

**Fig. 4:**
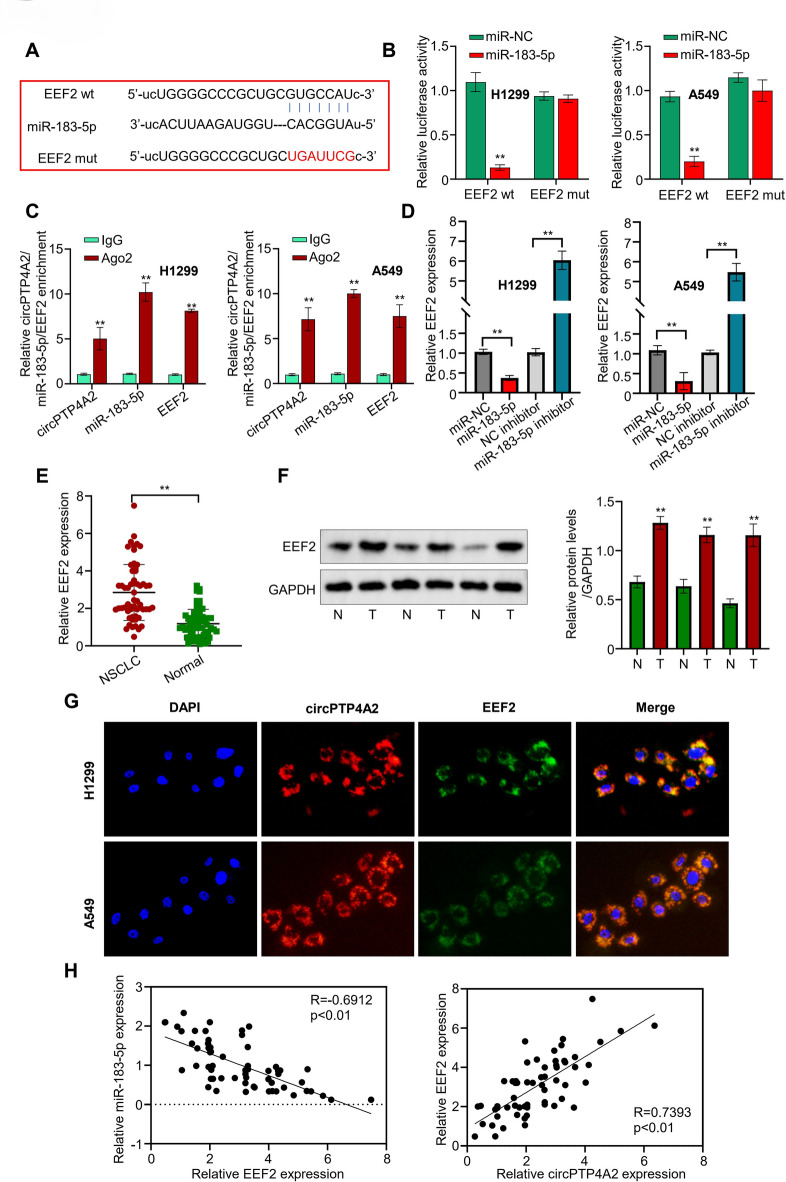
EEF2 is the target gene of miR-183-5P. **a** miR-183-5p and its putative binding sequence in the 3’-UTR of *EEF2*. **b** The luciferase activity of EEF2 was evidently inhibited by miR-183-5p. **c** RIP assay was performed to analyze the interaction between miR-183-5p and circPTP4A2, or EEF2. **d** qRT-PCR analysis of the mRNA expression of EEF2 in H1299 and A549 cells lines. **e** qRT-PCR analysis of EEF2 expression in lung cancer tissues. **f** EEF2 protein expression in NSCLC tissues was detected by western blot analysis. **g** The co-localization of circPTP4A2 (green, detected by FISH) and EEF2 (red, detected by IF) was observed in H1299 and A549 cells (magnification, × 200, Scale bar, 50 μm) by FISH assay. **h** EEF2 expression negatively correlates with miR-183-5p expression in NSCLC tissues and positively correlates with circPTP4A2 expression in NSCLC tissues. *N* = 3. **P < 0.01.

We subsequently aimed to identify the downstream target genes of miR-183-5p. Bioinformatics analysis using the Starbase database predicted a putative miR-183-5p binding site motif within the 3’UTR of *EEF2* (Fig. 4A). To empirically validate this interaction, we conducted dual-luciferase reporter plasmids containing either the wild-type or mutant 3’UTR of EEF2. The relative luciferase activity was significantly decreased in H1299 and A549 cells co-transfected with the wild-type reporter and miR-183-5p mimics, whereas this suppressive effect was abolished in cells co-transfected with the mutant vectors (Fig. 4B, *P* < 0.01). RIP assays revealed that circPTP4A2, miR-183-5p, and EEF2 were co-precipitated by the anti-Ago2 antibody (Fig. 4C, *P* < 0.01). Moreover, transfection with miR-183-5p mimics significantly suppressed EEF2 transcription, whereas miR-183-5p inhibitors elevated its expression levels (Fig. 4D, *P* < 0.01). Furthermore, qRT-PCR and Western blot analyses indicated that EEF2 expression in NSCLC specimen was markedly upregulated compared to adjacent normal tissues (Fig. 4E and F, *P* < 0.01). Before exploring the functional relationship between circPTP4A2 and EEF2, we assessed their subcellular distribution in H1299 and A549 cells using combined FISH assay and IF staining. The results displayed a distinct colocalization of circPTP4A2 (green) and EEF2 (red) predominantly within the cytoplasm (Fig. 4G). Additionally, correlation analysis revealed that EEF2 expression was inversely correlated with miR-183-5p abundance in NSCLC tissues, while exhibiting a positive correlation with circPTP4A2 levels (Fig. 4H, *P* < 0.01). Collectively, these findings suggest that EEF2 serves as a downstream target for miR-183-5p, and that circPTP4A2 is intricately involved in modulating EEF2 expression.

### CircPTP4A2 promotes NSCLC progression via miR-183-5p/EEF2 axis

**Fig. 5:**
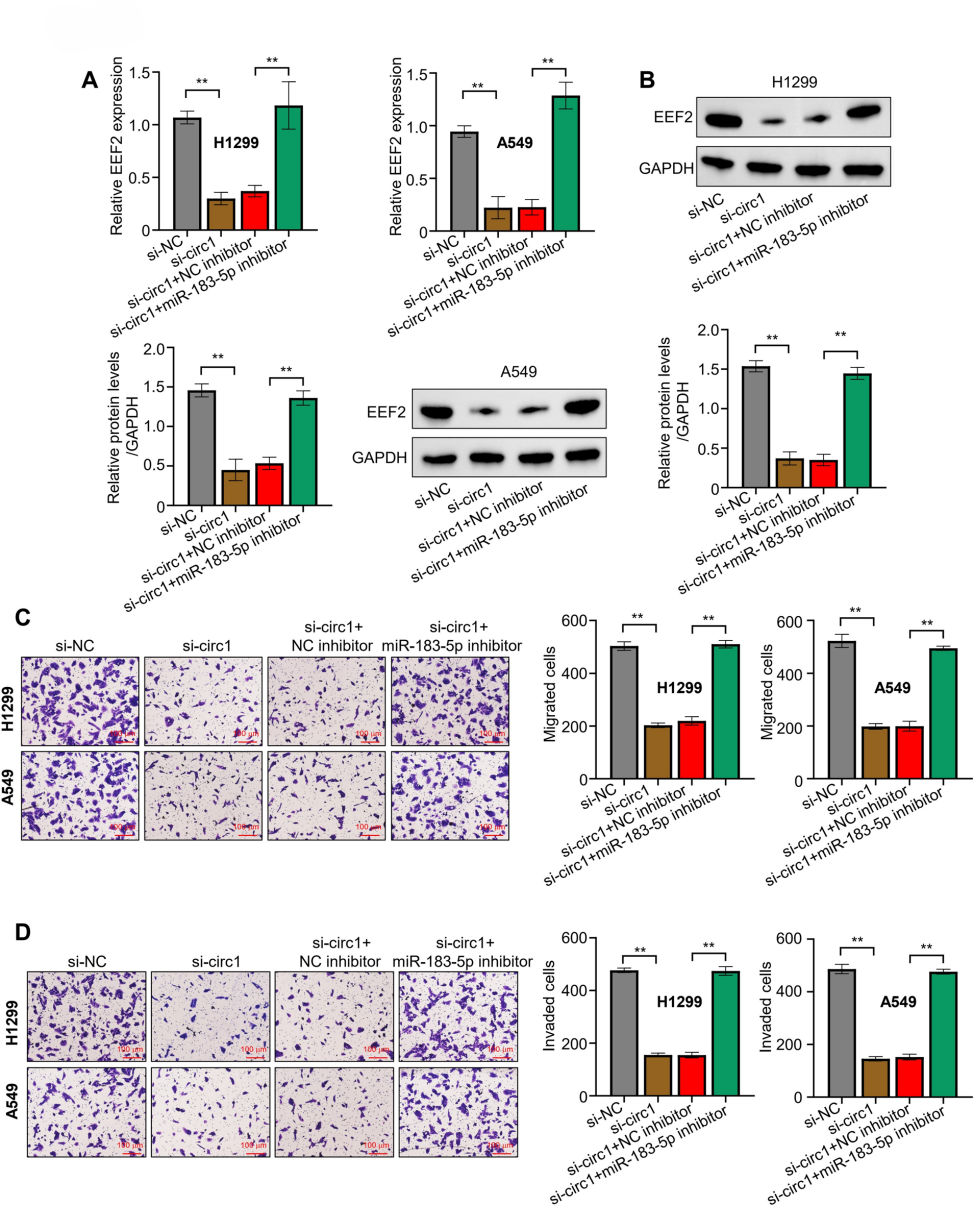
CircPTP4A2 promotes tumorigenesis and progression of NSCLC via miR-183-5P/EEF2 axis. **a** qRT-PCR analysis of the mRNA expression of EEF2 in H1299 and A549 cells lines. **b** Western blot analysis of the protein expression of EEF2 in H1299 and A549 cells lines. **c** and **d** Migration and invasion in treated H1299 and A549 cells lines were detected by Transwell assays (×200 magnification). *N* = 3. **P < 0.01.

We subsequently explored whether circPTP4A2 facilitates NSCLC progression via the miR-183-5p/EEF2 axis. Initially, H1299 and A549 cells were transfected with si-NC, si-circ1, or a combination of si-circ1 and miR-183-5p inhibitors. We observed that circPTP4A2 knockdown markedly decreased EEF2 transcription, an effect that was reversed by the introduction of miR-183-5p inhibitors (Fig. 5A and B, *P* < 0.01). Furthermore, silencing circPTP4A2 substantially impaired the migration and invasion capabilities of both cell lines (Fig. 5C and D, *P* < 0.01), however, these phenotypic changes were largely abrogated by miR-183-5p inhibition (Fig. 5C and D, *P* < 0.01).

## Discussion

For decades, circRNAs were largely regarded as transcriptional noise within eukaryotes due to their apparent lack of protein-coding capacity^24,25^. Nevertheless, the ubiquitous expression of circRNAs has since been identified across diverse cell types and species^25^. Furthermore, mounting evidence highlights that these circular transcripts exert critical functions in tumor progression and oncogenesis^26,27^.

The PRL subfamily of protein tyrosine phosphatases (PTPs), also known as PTP4A, consists of three highly homologous members, PRL1, PRL2, and PRL3 (encoded by the *PTP4A1*, *PTP4A2*, and *PTP4A3* genes, respectively), which share roughly 75% amino acid sequence identity. Recent investigations have revealed that PTP4A2 is essential for regulating the self-renewal capability and proliferation of hematopoietic stem cells^28^. The link between PRLs and oncogenesis initially garnered significant interest after PTP4A2 was found to be specifically overexpressed in various malignancies^29^. Consequently, the circPTP4A2-mediated oncogenic mechanism elucidated in our study, derived from the *PTP4A2* host gene, offers a novel and complementary perspective regarding the multifaceted roles of this phosphatase family in a broader array of human cancers. Structurally, circPTP4A2 is generated via the head-to-tail back-splicing and subsequent RNA circularization of two specific exons from the *PTP4A2* transcript^30,31^. Previous reports indicate that circPTP4A2 is upregulated in cervical cancer, where it augments tumor cell proliferation and invasiveness by modulating the miR-101-5p/MAT2A axis^32^. To elucidate the functional involvement of circPTP4A2 in NSCLC, qRT-PCR was employed to quantify its expression across 60 paired NSCLC tissues and adjacent non-tumor tissue specimens. Our findings revealed that circPTP4A2 expression is markedly elevated in NSCLC tissues compared to their normal counterparts. Furthermore, functional assays demonstrated that circPTP4A2 facilitates cell proliferation, migration, and invasiveness. These results indicate that circPTP4A2 actively drives NSCLC progression and serve as a promising therapeutic target for clinical intervention.

Mechanistically, it is well established that circRNAs modulate such cellular dynamics at both transcriptional and post-transcriptional levels^33^. For instance, circRNAs can bind to RNA binding proteins (RBPs), functioning as protein decoys to modulate their biological activities^25^. Additionally, while less common, certain circRNAs have been reported to govern gene expression at the transcriptional level, or even possess translation potential, as demonstrated by Perriman and Ares who engineered a translatable circular RNA encoding GFP in *E. coli*^34^. Moreover, circRNAs packaged within extracellular vesicles can be internalized by neighboring or distant cells. This intercellular transfer alters the pathophysiological states of recipient cells, thereby facilitating intercellular communication and driving tumor metastasis^35^. Furthermore, specific nuclear cirRNAs can associate with U1 SnRNP via RNA-RNA interactions, these complexes subsequently recruit RNA polymerase II to the promoter region of their host genes, thereby regulating host gene transcription^36^. Most commonly, however, circRNAs function as competitive endogenous RNAs (ceRNAs) by acting as miRNA sponge^37,38^. To elucidate the underlying mechanisms driving circPTP4A2-mediated NSCLC progression, we utilized the CircInteractome database to predict potential miRNA targets, revealing that the circPTP4A2 sequence harbors a putative binding site for miR-183-5p, leading to the upregulation of EEF2 expression and ultimately enhancing tumor cell proliferation and invasiveness. These observed oncogenic properties align with previous reports indicating that circPTP4A2 is frequently upregulated across various human malignancies, including NSCLC^19,39,40^. Further rescue experiments revealed that silencing miR-183-5p effectively reverses the inhibitory effects of circPTP4A2 knockdown on cellular migration and invasion. Taken together, our findings demonstrate that circPTP4A2 functions as a ceRNA by sponging miR-183-5p to upregulate the oncogene EEF2, thereby driving the progression of NSCLC. Although the present study establishes the oncogenic role of the circPTP4A2/miR-183-5p/EEF2 regulatory axis in NSCLC, the upstream mechanisms governing circPTP4A2 biogenesis and expression remain to be elucidated. The biogenesis of circular RNAs is intrinsically linked to the RNA polymerase II-mediated transcription of their parental genes, with circRNA abundance typically demonstrating a positive correlation with host genes transcriptional activity. Consequently, transcription factors orchestrating the expression of host gene PTP4A2 are highly likely to act as upstream regulators of circPTP4A2. Building upon the established transcriptional regulatory network of PTP4A2, we hypothesize that specific transcription factors driving the host gene also govern the biogenesis and expression of circPTP4A2. However, these hypotheses warrant further experimental validation in future investigations, representing a notable limitation of the current study. Additionally, a limitation of our current study is the relatively small clinical sample size (*n* = 60) and the limited number of OS events. This restricts the events per variable (EPV) ratio in our multivariate Cox regression model, which may marginally affect the statistical power. However, standard diagnostics indicated that the model remained stable and reliable. Future large-scale, multicenter cohort studies are warranted to further validate the prognostic value of circPTP4A2 in NSCLC. Furthermore, the lack of complete data regarding driver mutation status and detailed post-operative adjuvant therapies in our cohort represents another limitation that should be addressed in future prospective studies.

In conclusion, our research elucidates the oncogenic function of circPTP4A2 in NSCLC, which is driven by its modulation of the miR-183-5p/EEF2 axis. To our knowledge, this is the first study to delineate the molecular mechanisms of circPTP4A2 in NSCLC, thereby highlighting it as a promising therapeutic target for this malignancy.

## Supplementary Information

Below is the link to the electronic supplementary material.

Supplementary Material 1

Supplementary Material 2

Supplementary Material 3

## Data Availability

The data that support the findings of this study are available from the corresponding author upon reasonable request.
