# Supplementary material for: CircPTP4A2 (hsa_circ_0007364) promotes growth and invasion of non-small cell lung cancer by regulating miR-183-5p/EEF2 axis
Source: Sci Rep. 2026 May 8;16:21113. doi: 10.1038/s41598-026-50751-4 (PMC13342306; doi:10.1038/s41598-026-50751-4)
Supplement: Supplementary file 1 — Supplementary Material 1 [file 41598_2026_50751_MOESM1_ESM.pdf]

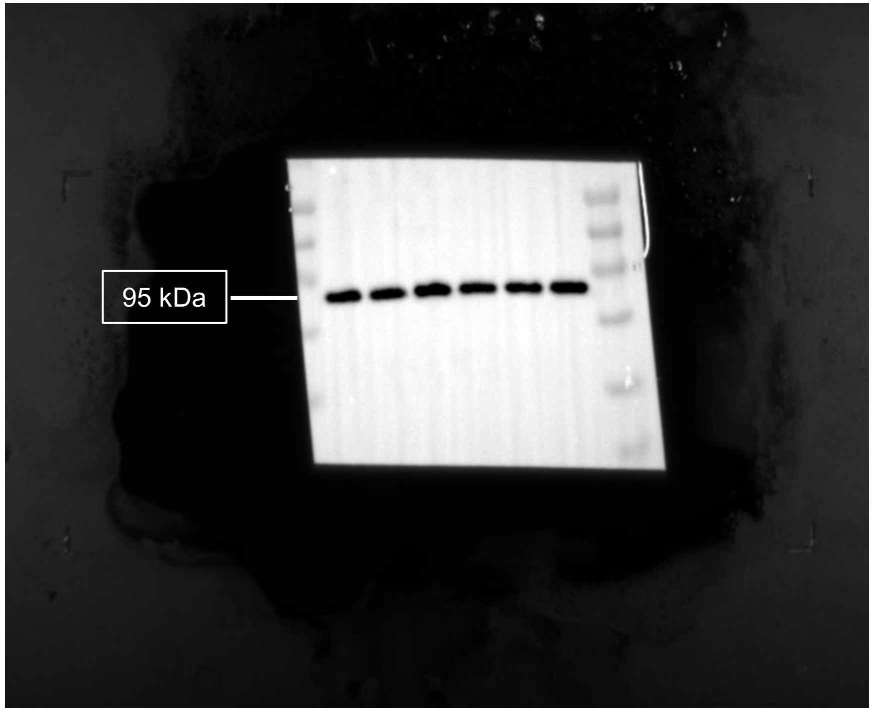

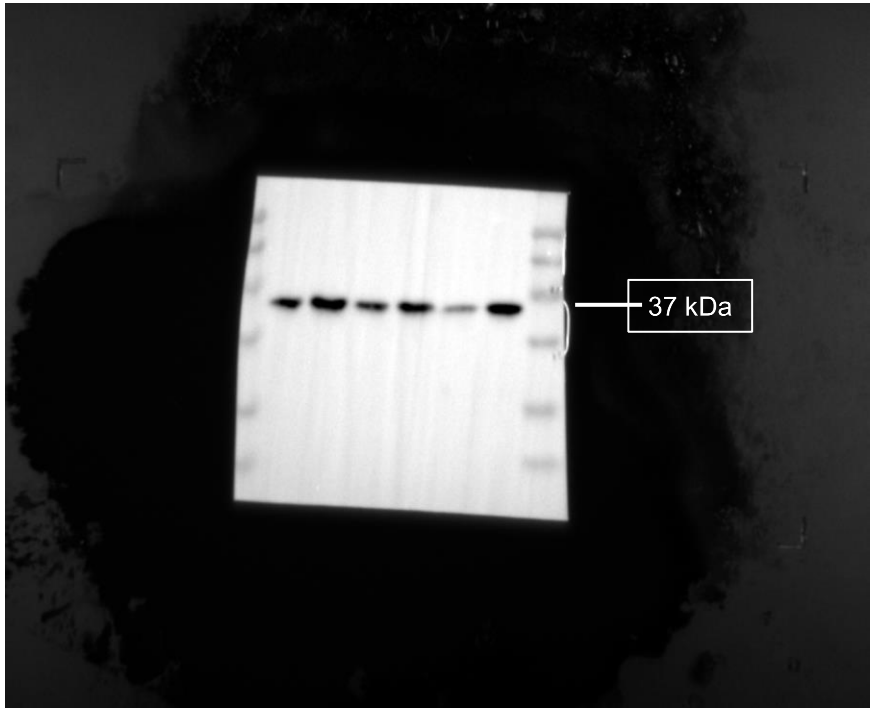

Raw data of blots for Figure 4F (upper panel in NSCLC tissues)

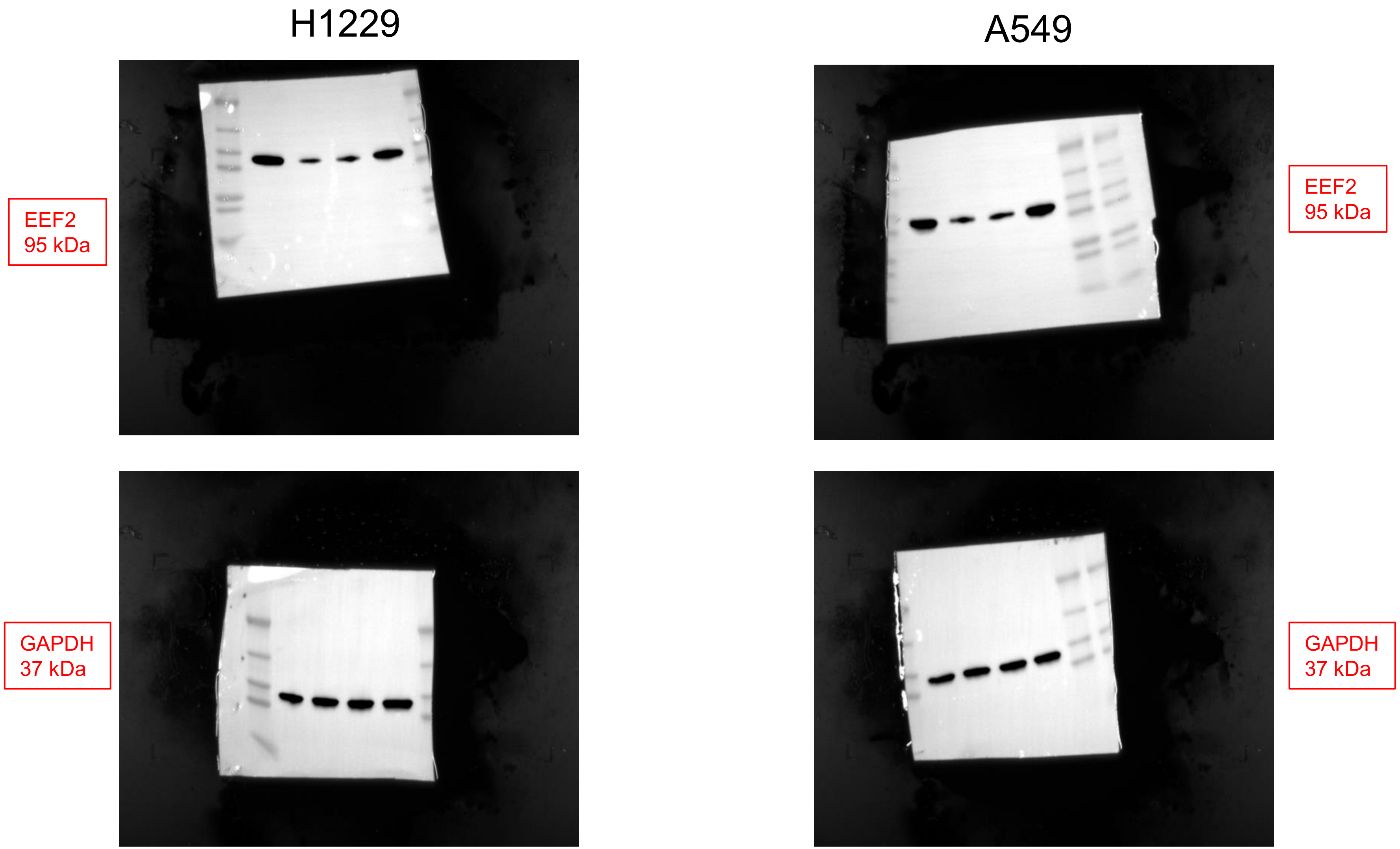

Raw data of blots for Figure 5B (upper panel in H1229 and A549 cells)

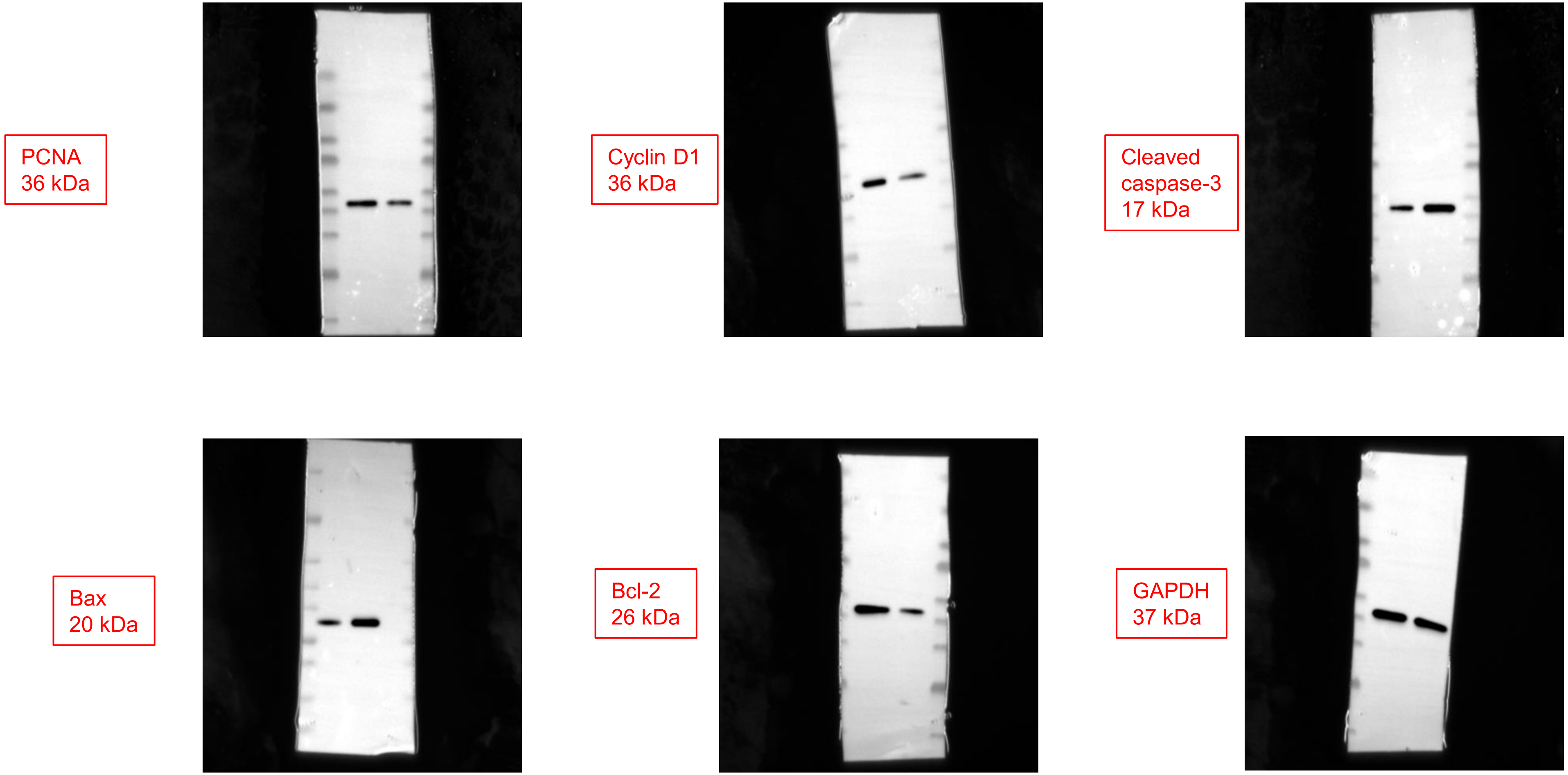

Raw data of blots for Figure S1
