# Supplementary material for: CircPTP4A2 (hsa_circ_0007364) promotes growth and invasion of non-small cell lung cancer by regulating miR-183-5p/EEF2 axis
Source: Sci Rep. 2026 May 8;16:21113. doi: 10.1038/s41598-026-50751-4 (PMC13342306; doi:10.1038/s41598-026-50751-4)
Supplement: Supplementary file 2 — Supplementary Material 2 [file 41598_2026_50751_MOESM2_ESM.docx]

**Table S1. Sequences of oligonucleotides used in this study.**

| **Name** | **Sequence (5’to 3’)** |
| --- | --- |
| si-NC | 5’-CAACAAGAUGAAGAGCACCAA-3’ |
| si-circPTP4A2-1 | 5’-GUUCUAGUUUUUCGUUGGAAU-3’ |
| si-circPTP4A2-2 | 5’-AGGCAUUUUAGTGGUCUUUUUAA-3’ |
| miR-183-5p mimics | 5’-UCACUUAAGAUGGUCACGGUAU-3’ |
| miR-NC mimics | 5’-UUCUCCGAACGUGUCACGUTT-3’ |
| miR-183-5p inhibitor | 5’-AGUGAAUUCUACCAGUGCCAUA-3’ |
| miR-NC inhibitor | 5’-CAGUACUUUUGUGUAGUACAA-3’ |
