# Supplementary material for: CircPTP4A2 (hsa_circ_0007364) promotes growth and invasion of non-small cell lung cancer by regulating miR-183-5p/EEF2 axis
Source: Sci Rep. 2026 May 8;16:21113. doi: 10.1038/s41598-026-50751-4 (PMC13342306; doi:10.1038/s41598-026-50751-4)

Figure S1. (A) Heatmap of differentially expressed circular RNAs according to GSE146689, GSE101586, and GSE112214 (Log2 FC >1, P < 0.05). Red indicates the upregulated circRNA in NSCLC. Blue indicates the downregulated circRNAs in NSCLC. (B) qRT-PCR shows that the level of circPTP4A2 in LV-circ1 transfected tumor is lower than that in LV-NC transfected tumor. (C) Proliferation- and apoptosis-related proteins expression in tumor was detected by western blot analysis. N=6. **P<0.01.


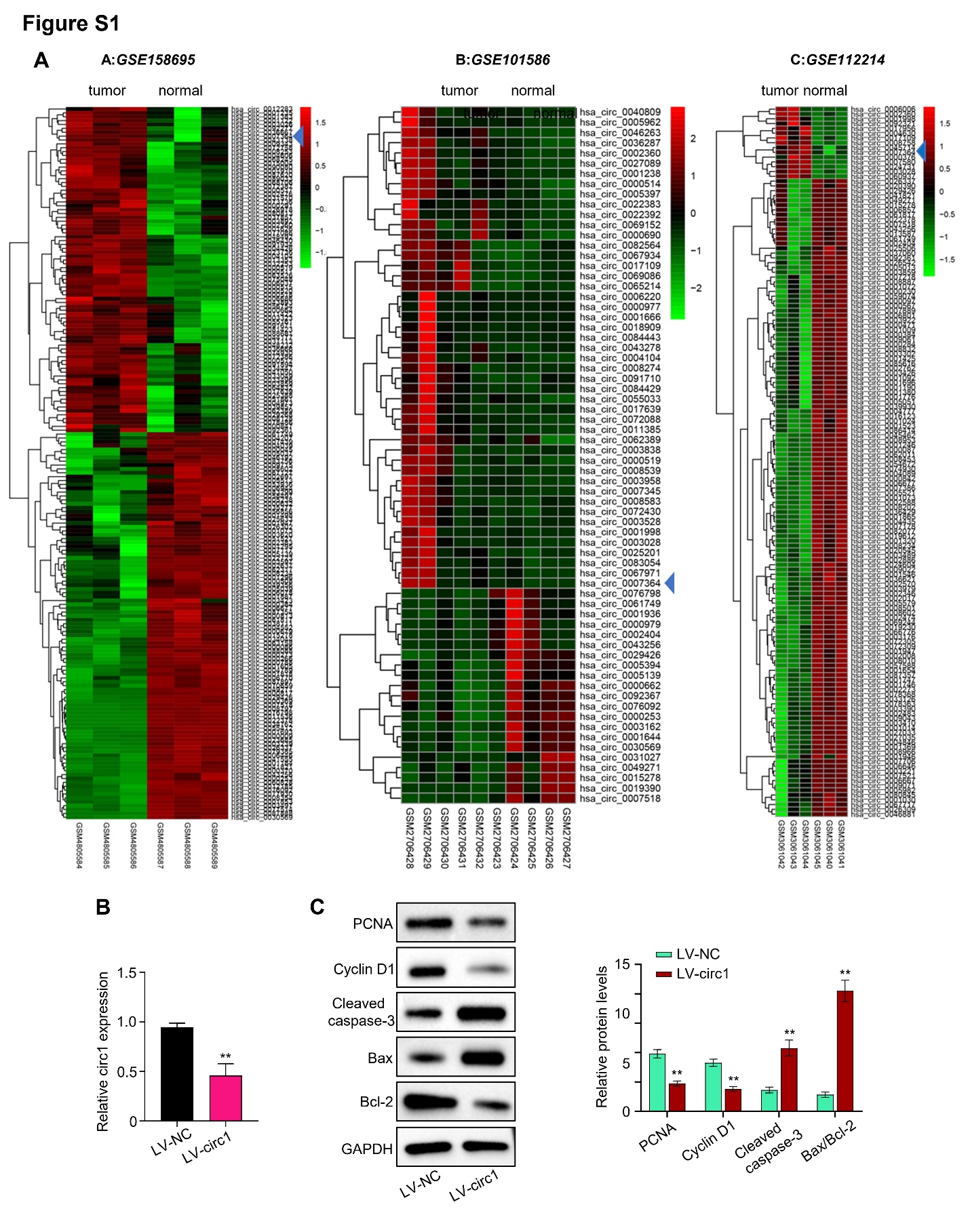

Supplement: Supplementary file 3 — Supplementary Material 3 [file 41598_2026_50751_MOESM3_ESM.docx]
